# Inter-study reproducibility of cardiovascular magnetic resonance myocardial feature tracking

**DOI:** 10.1186/1532-429X-14-43

**Published:** 2012-06-21

**Authors:** Geraint Morton, Andreas Schuster, Roy Jogiya, Shelby Kutty, Philipp Beerbaum, Eike Nagel

**Affiliations:** 1 King's College London British Heart Foundation (BHF) Centre of Excellence; National Institute of Health Research (NIHR) Biomedical Research Centre at Guy's and St. Thomas' NHS Foundation Trust; Wellcome Trust and Engineering and Physical Sciences Research Council (EPSRC) Medical Engineering Centre; Division of Imaging Sciences and Biomedical Engineering, The Rayne Institute, 4th Floor Lambeth Wing, St. Thomas' Hospital, London, SE1 7EH, United Kingdom; 2 Joint Division of Pediatric Cardiology, University of Nebraska College of Medicine/ Creighton University School of Medicine, Children’s Hospital and Medical Center, Omaha, NE, USA; 3 Departments for Radiology and Paediatric Cardiology, St Radboud Medical University, Nijmegen, The Netherlands

## Abstract

**Background:**

Cardiovascular magnetic resonance myocardial feature tracking (CMR-FT) is a recently described method of post processing routine cine acquisitions which aims to provide quantitative measurements of circumferentially and radially directed ventricular wall strain. Inter-study reproducibility is important for serial assessments however has not been defined for CMR-FT.

**Methods:**

16 healthy volunteers were imaged 3 times within a single day. The first examination was performed at 0900 after fasting and was immediately followed by the second. The third, non-fasting scan, was performed at 1400.

CMR-FT measures of segmental and global strain parameters were calculated. Left ventricular (LV) circumferential and radial strain were determined in the short axis orientation (Ecc_SAX_ and Err_SAX_ respectively). LV and right ventricular longitudinal strain and LV radial strain were determined from the 4-chamber orientation (Ell_LV_, Ell_RV_, and Err_LAX_ respectively). LV volumes and function were also analysed.

Inter-study reproducibility and study sample sizes required to demonstrate 5% changes in absolute strain were determined by comparison of the first and second exams. The third exam was used to determine whether diurnal variation affected reproducibility.

**Results:**

CMR-FT strain analysis inter-study reproducibility was variable. Global strain assessment was more reproducible than segmental analysis. Overall Ecc_SAX_ was the most reproducible measure of strain: coefficient of variation (CV) 38% and 20.3% and intraclass correlation coefficient (ICC) 0.68 (0.55-0.78) and 0.7 (0.32-0.89) for segmental and global analysis respectively. The least reproducible segmental measure was Ell_RV_: CV 60% and ICC 0.56 (0.41-0.69) whilst the least reproducible global measure was Err_LAX_: CV 33.3% and ICC 0.44 (0–0.77). Variable reproducibility was also reflected in the calculated sample sizes, which ranged from 11 (global Ecc_SAX_) to 156 subjects (segmental Ell_RV_). The reproducibility of LV volumes and function was excellent. There was no diurnal variation in global strain or LV volumetric measurements.

**Conclusions:**

Inter-study reproducibility of CMR-FT varied between different parameters, as summarized above and was better for global rather than segmental analysis. It was not measurably affected by diurnal variation. CMR-FT may have potential for quantitative wall motion analysis with applications in patient management and clinical trials. However, inter-study reproducibility was relatively poor for segmental and long axis analyses of strain, which have yet to be validated, and may benefit from further development.

## Background

Myocardial wall motion analysis is key for assessment of left ventricular (LV) and right ventricular (RV) function. It is important for the evaluation of cardiomyopathies [1] and for identification of hibernation and ischaemia during dobutamine stress in patients with coronary artery disease (CAD) [2]. Cardiovascular magnetic resonance (CMR) is an established method for wall motion assessment as it provides excellent endocardial border definition and can be combined with other techniques for a comprehensive, radiation-free, cardiac assessment in a single session [3]. Currently visual assessment of wall motion is standard however this is limited by inter-observer variability [4]. To date quantitative techniques for assessment of wall motion, such as myocardial tagging, have not been widely adopted due to the requirement to perform additional scans and the associated complex and time-consuming post-processing [5,6]. Alternative methods, which allow robust and user-independent quantitative wall motion assessment, are therefore desirable.

CMR myocardial feature-tracking (CMR-FT) has been recently described [7,8] and is a method of quantitative wall motion assessment which is analogous to echocardiographic speckle-tracking. Standard CMR cine images are analysed off-line and acquisition of additional sequences is therefore not required. CMR-FT aims to provide measurements of circumferentially, radially and longitudinally directed ventricular wall strain. However, in order to be robust and useful, analytical techniques must be sufficiently reproducible. Reasonable inter and intra-observer reproducibility of feature tracking has already been demonstrated [9,10]. The aim of this study therefore was to evaluate the inter-study reproducibility of CMR-FT and ascertain whether this is affected by detectable physiological variation.

## Methods

### Population

16 healthy volunteers were recruited by emailing university staff and students. Exclusion criteria were: known cardiac, respiratory or renal disease or a contraindication to MRI. The local ethics committee approved the study and all participants gave written informed consent.

### Data acquisition

Participants underwent 3 CMR examinations on the same day. All imaging was performed on a 3 Tesla clinical MRI scanner (Achieva, Philips Medical Systems, Best, The Netherlands), with participants supine, and using a 32-channel phased array receiver cardiac coil. On the day of the scans subjects were asked to fast from midnight. The first CMR examination (exam A) was performed at 0900, and the second (exam B) immediately afterwards at 0930. In order to try and maximise physiological changes participants then left the department and ate and drank as normal. They returned at 1400 for the third scan (exam C). Exams A and B were acquired to assess for the inherent variability of CMR-FT and exams A and C to assess for additional physiological changes due to circadian rhythms, and changes in hydration.

As part of the protocol scans included an initial survey, coil reference scan and planning to define imaging planes. Cine images were acquired using a standard balanced steady state free precession (SSFP) sequence. Planning and acquisition were as follows: A pseudo 2-chamber cine was planned from the axial survey images and this was subsequently used to plan a pseudo 4-chamber cine. Systolic frames from the pseudo 2 and 4-chamber cines were then used to plan 3 short axis cine slices of the basal, mid and apical LV. Diastolic frames from preceding cines were then used to plan definitive views. The pseudo 2-chamber and short axis views were used to plan the 4-chamber cine. This 4-chamber view and the pseudo short axis slices were used to plan the 2-chamber cine. The 2 and 4-chamber views were used to plan, contiguous short axis (SA) cine slices covering the entire LV, and the 4-chamber and short axis views to plan a 3-chamber cine. SSFP cine in-plane resolution was 1.8 x 2 mm and slice thickness 8 mm. The protocol was the same for all 3 scans and for all participants. Participants were removed from the scanner between each exam. Each scan lasted approximately 30-minutes.

### Data analysis

#### Feature tracking

Images were analysed in a random order by a blinded experienced observer. CMR-FT strain analysis was performed using dedicated prototype software (Diogenes MRI, Tomtec, Unterschleissheim, Germany). Endocardial contours were manually drawn in all views included in the analysis. The 4-chamber view was used to determine RV and LV longitudinal strain and LV radial strain (Ell_RV_ and Ell_LV_ and Err_LAX_). LV short axis circumferential (Ecc_SAX_) and radial strains (Err_SAX_) were calculated from a mid-ventricular short-axis view containing both papillary muscles. The RV upper septal insertion point was manually defined to allow accurate segmentation according to a recognized standard model [11]. All parameters were analysed for all three exams. Strain values (% change from baseline) were obtained for each segment and global values defined as the mean of all segmental values.

#### Left ventricular volumes and function

Images were analysed in a random order by a blinded experienced observer using CMR42 (Circle, Calgary, Canada). The mitral valve plane and apex were identified from the 4-chamber view in end-diastole and end-systole. The LV endocardial border was automatically defined in the corresponding end-systolic and end-diastolic SA slices and was manually corrected where required. Papillary muscles were excluded.

#### Statistical analysis

Statistical analysis was performed using Microsoft Excel and IBM SPSS Statistics version 19. For inter-study reproducibility evaluation a coefficient of variation (CV), intraclass correlation coefficient (ICC) and Bland Altman plots were calculated. For each parameter the mean value and differences between exam A and B and exam A and C values were calculated. The CV was defined as the standard deviation of the differences divided by the mean [12]. Analyses were all performed on a per observation basis.

The significance of differences in reproducibility between exam A and B (reproducibility AB) and exam A and C (reproducibility AC) was evaluated by comparing the squared differences between the observations. These were compared directly with a paired t test if normally distributed (LV volumes and function) or after natural log transformation if not normally distributed (strain) [12]. The Shapiro-Wilk test determined normality. The mean values for each parameter from exam A, B and C were not normally distributed and therefore were compared using the Friedman test.

Study sample sizes required to show a 5% absolute change in strain with a power of 90% and an α error of 0.05 were calculated as follows [12]:

(1) n=fα,P.σ2.2δ2

where n is the sample size, f =10.5 for α 0.05 and P 0.9, σ the inter-study standard deviation and δ the magnitude of the difference to be detected.

Continuous data are presented as mean ± standard deviation. Significance was determined as p < 0.05.

## Results

### Participant details

Participant age was 27.9 ± 5.7 and body mass index 26.2 ± 6.8. Eight were male and 8 female. One participant chose not to re-attend for scan C after scans A and B. All other participants completed the protocol. The CMR data of one other participant was found to be incompatible with the CMR-FT software (transient high signal in the pulmonary arteries affected the grey scaling obscuring endocardial border tracking). This participant was therefore only included in the volumetric assessments. Image quality was otherwise good or excellent in all subjects.

### Feature tracking

Mean segmental and global strain parameters for all three exams are summarised in Table 1. Mean segmental Err_SAX_ was significantly different (p = 0.04) between the scans although the largest difference was between scans A and B. There were no significant differences in any of the other strain parameters.

**Table 1:** Mean ± standard deviation segmental and global strain values for the combined study population

	**Exam**			**p**
	**A**	**B**	**C**	
**Segmental**				
Err_SAX_	22.4 ± 9.6	19.2 ± 8.0	20.9 ± 9.0	0.04*
Ecc_SAX_	−17.6 ± 8.1	−16.6 ± 8.4	−18.2 ± 8.9	0.06
Err_LAX_	16.2 ± 10.2	17.7 ± 10.3	17.9 ± 10.5	0.25
Ell_LV_	−21.0 ± 11.0	−20.0 ± 11.0	−19.2 ± 12.8	0.23
Ell_RV_	−23.7 ± 16.4	−21.7 ± 13.5	−20.6 ± 14.7	0.25
**Global**				
Err_SAX_	22.6 ± 7.9	19.4 ± 6.7	20.5 ± 5.2	0.40
Ecc_SAX_	−17.6 ± 5.0	−16.6 ± 4.4	−18.1 ± 4.3	0.81
Err_LAX_	16.2 ± 5.6	17.7 ± 5.6	17.9 ± 3.8	0.29
Ell_LV_	−21.0 ± 5.1	−20.0 ± 5.3	−19.2 ± 5.3	0.06
Ell_RV_	−23.8 ± 9.9	−21.8 ± 5.7	−19.1 ± 8.7	0.26

Values are in %. p values refer to the significance of differences between the mean values from exams A, B and C. *denotes significant differences.

#### Inter-study reproducibility

**Figure 1:**
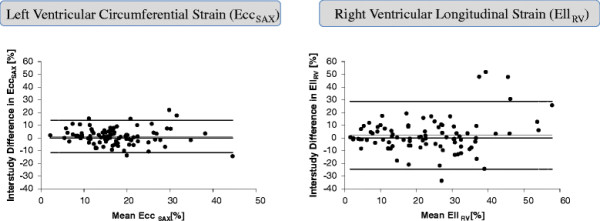
**Inter-study agreement of segmental strain as determined by feature tracking.** Bland Altman plots with limits of agreement (95% confidence intervals) demonstrating the inter-study reproducibility of CMR myocardial feature tracking segmental left ventricular circumferential and right ventricular longitudinal strain.

CV, ICCs and sample size calculations for each strain parameter are summarised in Table 2. Reproducibility was highly variable. Global strain assessment was more reproducible than segmental analysis. Overall Ecc_SAX_ was the most reproducible measure of strain: CV 38% and 20.3% and ICC 0.68 (0.55-0.78) and 0.7 (0.32-0.89) for segmental and global analysis respectively. The least reproducible segmental measure was Ell_RV_: CV 60% and ICC 0.56 (0.41-0.69) whilst the least reproducible global measure was Err_LAX_: CV 33.3% and ICC 0.44 (0–0.77). Bland-Altman plots demonstrate the reproducibility of segmental Ecc_SAX_ and Ell_RV_ (Figure1).

**Table 2:** Segmental and global inter-study reproducibility for five different feature tracking strain parameters and the study sample size required to detect an absolute change of 5% with 90% power and an α error of 0.05

	**Mean difference ± SD**	**Coefficient of Variation**	**ICC (95%CI)**	**Sample size**
**Segmental**				
Err_SAX_	−3.5 ± 7.5%	35.9%	0.57 (0.35-0.71)	48
Ecc_SAX_	1.2 ± 6.5%	38.0%	0.68 (0.55-0.78)	36
Err_LAX_	1.5 ± 9.2%	53.2%	0.62 (0.47-0.73)	72
Ell_LV_	−1.0 ± 11.3	55.4%	0.59 (0.44-0.71)	108
Ell_RV_	−1.9 ± 13.6	60.0%	0.56 (0.41-0.69)	156
**Global**				
Err_SAX_	−3.2 ± 5.7%	27.2%	0.61 (0.15-0.85)	28
Ecc_SAX_	1.0 ± 3.5%	20.3%	0.70 (0.32-0.89)	11
Err_LAX_	1.5 ± 5.8%	33.3%	0.44 (0–0.77)	29
Ell_LV_	−1.08 ± 5.4%	26.4%	0.44 (0–0.77)	25
Ell_RV_	−1.9 ± 6.8%	29.9%	0.62 (0.20-0.85)	39

*SD* = Standard deviation; *ICC* = Intraclass correlation coefficient; *CI* = Confidence Interval

Sample size numbers must be doubled for studies comparing active treatment vs. placebo.

This variable reproducibility is reflected in the sample size calculations. 5% changes in absolute global strain can be detected in follow-up studies with relatively few patients whilst many more are required for some segmental strain parameters. Sample sizes for global strain parameters ranged from 11 (Ecc_SAX_) to 39 (Ell_RV_) and for segmental strain from 36 (Ecc_SAX_) to 156 (Ell_RV_).

#### Comparison of inter-study reproducibility AB with AC

Inter-study reproducibility AC was not significantly different from reproducibility AB for any segmental or global parameters (p > 0.05 for all).

### LV volumes and function

#### Inter-study reproducibility

Mean LV volumes and function are shown in Table 3. There were no significant differences in LV volumes or function between the three exams. Inter-study reproducibility was excellent overall. CV and ICC for LV end-diastolic volume, end-systolic volume and ejection fraction were 4.2% and 0.98; 8.3% and 0.95; and 4.6% and 0.75 respectively (sssTable 4).

**Table 3:** Mean left ventricular volumes and function for the entire study population

	**Exam**			**p**
	**A**	**B**	**C**	
**LVEDV**	161.7 ± 33.3 ml	162.5 ± 37.0 ml	161.2 ± 39.7 ml	0.76
**LVESV**	67.5 ± 17.3 ml	68.9 ± 20.0 ml	64.9 ± 18.3 ml	0.28
**LVEF**	58.5 ± 3.2%	58.0 ± 4.3%	59.6 ± 4.5%	0.86

*p* values refer to the significance of differences between exam A, B and C

*LVEDV* = left ventricular end-diastolic volume; *LVESV* = left ventricular end-systolic volume; *LVEF* = left ventricular ejection fraction.

**Table 4:** Inter-study reproducibility for left ventricular volumes and function

	**Mean difference ± SD**	**Coefficient of Variation**	**Intraclass correlation coefficient (95%CI)**
**LVEDV**	−0.4 ± 6.9 ml	4.2%	0.98 (0.97-1.0)
**LVESV**	−0.8 ± 5.3 ml	8.3%	0.95 (0.88-0.98)
**LVEF**	0.3 ± 2.6 ml	4.6%	0.75 (0.43-0.91)

*LVEDV* = left ventricular end-diastolic volume; *LVESV* = left ventricular end-systolic volume; *LVEF* = left ventricular ejection fraction; *CI* = confidence interval.

#### Comparison of inter-study reproducibility AB with AC

There were no significant differences between reproducibility AB and AC for LV volumes or function (p > 0.05 for all).

## Discussion

This study demonstrates that the inter-study reproducibility of CMR-FT parameters is highly variable and is better for global rather than segmental measures. Ecc_SAX_ is the most reproducible whilst others such as Ell_RV_ and Err_LAX_ are less reproducible. Furthermore there was no measurable diurnal variation.

### CMR feature tracking

Abnormalities or inter-study changes in myocardial strain can occur without any detectable changes in LV volumes or function. This has been demonstrated, for example, in studies examining the treatment effect of stem cells in myocardial infarction [13,14]. Myocardial strain imaging may therefore allow early detection of abnormalities and subsequently allow early diagnosis and intervention. However despite this imaging techniques for strain analysis have not been widely adopted. CMR-FT has been described in detail previously [8] and appears to be a promising new tool for quantitative assessment of wall motion analysis. Borders within the image, such as the border between the LV endocardium and LV blood, are identified and then tracked from frame to frame. This relies on quantification of changes in signal intensity within voxels from one frame to the next. Frame-to-frame displacement of these signal changes is used to calculate local myocardial velocity and deformation or strain.

The accuracy of CMR-FT is likely to depend on the orientations and dimensions of the relevant border or features relative to voxels of the slice. Furthermore CMR- FT software derives strain measurements from the apparent in-plane movements. However the apparent movements of features in 2D cine images may be caused by through-plane displacements of oblique or tapering structures, and not only by in-plane movements. This potential limitation of the technique may explain some of the variability seen in reproducibility in this study.

Global Ecc_SAX_ from the mid LV slice has been shown to correlate strongly with myocardial tagging using Harmonic Phase Imaging (HARP) in patients with Duchene Muscular Dystrophy and varying LV dysfunction and also in normal volunteers [15]. Ecc_SAX_ has also been compared with a complementary spatial modulation of magnetization (CSPAMM) myocardial tagging reference method in a subsequent study [16]; agreement was modest and notably segmental strain measurements were less good than global Ecc_SAX_ measurements. However, to date, other CMR-FT parameters have not been validated against a reference standard for the evaluation of myocardial strain. Further validation of CMR-FT strain measurements, particularly segmental measurements, is therefore desirable.

### Inter-study reproducibility

Inter-study reproducibility of a technique is key where repeated examinations are required. The absence of ionising radiation means that CMR lends itself well to repeated testing. Wall motion assessment with CMR-FT could therefore potentially be useful for serial examinations of ventricular function to assess disease progression, treatment efficacy or determine the timing of therapy. Serial examinations are important in a multitude of clinical conditions and also form a key component of many studies. Higher reproducibility means that smaller changes can be detected with increased reliability. This also results in cost-efficiencies, as fewer subjects are required in clinical trials [17].

Our sample size calculations demonstrate that relatively small sample sizes are required to detect 5% changes in global strain parameters, particularly Ecc_SAX_. However, a 5% change might be a relatively large effect, and larger sample sizes would be required if subtle changes in strain were likely to be clinically relevant. Furthermore, lower reproducibility of some segmental strain parameters means that large sample sizes would be required to detect differences in these. This is likely to be particularly relevant for assessing patients with CAD when regional analysis is crucial. Therefore whilst this new technique is promising for quantitative wall motion assessment its utility would be greater if further method developments improved reproducibility.

To our knowledge this is the first assessment of CMR-FT inter-study reproducibility. However, previous studies by our group have suggested that inter and intra observer reproducibility is higher for Ecc_SAX_ than for Err_SAX_ both in volunteers and in patients with ischaemic cardiomyopathy [9,10]. Inter-study variability was of a similar magnitude to the intra-observer and inter-observer variability reported previously although intra and inter-observer reproducibility may be superior to inter-study reproducibility.

This study included normal volunteers and image quality was good to excellent in all cases. Reproducibility and sample sizes may therefore be different in patient studies or when image quality is reduced. However, in previous studies of volunteers [9] and patients with ischaemic cardiomyopathy [10], CMR-FT intra- and inter-observer reproducibility were no worse during low-dose dobutamine infusion than at rest, even though dobutamine is often associated with a deterioration in image quality. Furthermore, CMR-FT reproducibility in patients with ischemic cardiomyopathy appeared to be comparable to that of healthy volunteers despite reduced LV function in the patients. These observations suggest that the findings of this study can be applied to patients. However, it is important to note that in the patient study 13% of segments were excluded, mainly due to breathing artefacts.

This study also confirms the excellent inter-study reproducibility of CMR for evaluating LV volumes and function demonstrated in previous studies [12,17]. In keeping with these studies, using CV as a measure of inter-study reproducibility, we also found LV end-systolic volume to be slightly less reproducible than both LV end-diastolic volume and LV ejection fraction. However, ICC was lower for ejection fraction than for LV volumes, emphasizing the fact that multiple measures of inter-study reproducibility exist and the potential difficulties in comparing results from studies using different statistical methods. We have reported multiple measures of reproducibility in order to be comprehensive.

### Physiological variation

Many biological processes exhibit circadian rhythms and heart rate and blood pressure changes are well known [18]. Consequently studies are often designed to minimise the effects of physiological variation either as a result of circadian rhythms or changes in participants’ volume status and therefore ventricular volumes [12]. However, data on whether detectable diurnal changes in left ventricular volumes and function actually occur are limited [19,20]. Previous echocardiographic studies have suggested that there are no diurnal variations in LV volumes and systolic function. Nonetheless variations in some parameters, of up to approximately 20%, have been demonstrated in healthy volunteers [19,21]. In keeping with these studies we did not detect any diurnal changes in LV volumes and function or in LV or RV myocardial strain. Furthermore, the inter-study reproducibility of LV volume and function and strain measurements was not measurably affected by diurnal variation. These findings suggest that diurnal variations are not currently important considerations when repeated studies are performed for CMR-FT and/or volumetric analysis. However, it is important to note that the higher variability of the strain measurements means that small changes are unlikely to have been detected. The sample size calculations suggest that our sample was large enough to detect a 5% change in Ecc_SAX_.

### Comparison with speckle tracking

Speckle tracking with echocardiography is a more established technique than CMR-FT. [22] Previous echocardiography based studies have shown the clinical utility of speckle tracking for LV viability assessment and planning of revascularisation, assessment of patients with acute myocardial infarction and heart failure with normal ejection fraction and as an early marker of LV dysfunction in patients with Tetralogy of Fallot [23-27]. Data on the inter-study reproducibility of strain analysis speckle tracking is limited and it is therefore difficult to draw comparisons with the CMR findings of this study. Reasonable inter-study reproducibility of left ventricular rotation parameters (as opposed to strain) has been demonstrated [28] using different measures of reproducibility.

Recently echocardiographic global longitudinal speckle strain was shown to be an independent predictor of mortality in a population of 546 consecutive patients undergoing echocardiography for left ventricular assessment [29]. Global longitudinal strain was a stronger predictor than both ejection fraction and wall motion scoring. This raises the possibility that the analogous global longitudinal LV feature tracking strain measured in this study, and possibly other strain parameters, may also be useful prognostic markers and our data show reasonably good reproducibility of global parameters. Larger scale long-term CMR studies are required to test this hypothesis.

Comparative studies between CMR-FT and speckle tracking have not yet been performed. Both techniques have potential advantages. Reliable endocardial border detection is key for strain imaging and superior image quality and endocardial border definition with CMR may be an advantage. Echocardiography has different axial and lateral resolutions and can also be affected by limited acoustic windows. Border tracking may therefore be more reliable with better image quality. Conversely although CMR provides relatively good contrast to noise at most epicardial and endocardial boundaries it may not be able to detect similar intra-myocardial features, and therefore intra-myocardial strain, as well as speckle tracking. CMR myocardial tagging imaging techniques may also be superior to CMR-FT in this respect. Moreover, echocardiography has superior temporal resolution, which may translate into an advantage for speckle tracking over CMR-FT.

### Limitations

The main limitations of this study are its small size and the inclusion of normal volunteers rather than patients. However it is common to evaluate emerging techniques in volunteers initially and these studies are important platforms for further method improvement and subsequent patient studies. Moreover limited previously published data suggest that volunteer and patient reproducibility may well be similar as discussed above.

## Conclusions

Inter-study reproducibility of CMR-FT is highly variable. At present reproducibility is best for global measurements and LV circumferential strain is the most reproducible strain parameter. CMR-FT strain analysis is not measurably influenced by diurnal variation, although this may be due to the small sample size. CMR myocardial strain analysis with feature tracking may have potential clinical and research applications. However the technique, particularly segmental and long axis analyses of strain, would benefit from improved inter-study reproducibility and also further validation studies.

## Competing interests

The authors declare that they have no competing interests.

## Authors’ contributions

GM and AS designed the study protocol, acquired and analysed the data and drafted the manuscript. RJ, SK and PB helped acquire and analyse the data and critically revised the manuscript. EN assisted with study design and interpretation of data and critically revised the manuscript. All authors read and approved the final manuscript.
